# ISBNPA 2007: Marketing, serious games and nanny states. Observations from the sixth annual meeting of the International Society of Behavioral Nutrition and Physical Activity, Oslo 2007

**DOI:** 10.1186/1479-5868-4-37

**Published:** 2007-09-19

**Authors:** Johannes Brug

**Affiliations:** 1 EMGO Institute, VU University Medical Center, Van der Boechorststraat 7; 1081 BT Amsterdam, The Netherlands

## Abstract

This commentary paper provides a selective overview of topics addressed at the sixth annual meeting of the International Society of Behavioral Nutrition and Physical Activity (ISBNPA). With 31 symposiums, 42 free paper sessions and 236 poster presentations ISBNPA 2007 provided a comprehensive overview of the state of the art and of new avenues for behavioral nutrition and physical activity research. Research presented at the conference helps to identify and specify important nutrition and physical activity behaviors for health promotion, as well as the correlates, predictors and determinants of these behaviors, and to build and test intervention strategies that go beyond traditional health education. ISBNPA 2007 also indicates that ISBNPA should strive to become more international by inclusion of more scientists from countries outside North America, Western Europe and Australia. ISBNPA should maintain its encouragement of research that is firmly rooted in behavioral theory and research that goes beyond applying cross-sectional research designs, and that addresses the most important public health issues associated with behavioral nutrition and physical activity.

## Background

The sixth annual meeting of the International Society of Behavioral Nutrition and Physical Activity was held in Oslo June 22 to 24, 2007. As president-elect I was responsible for the program and therefore busy with organizational issues during the conference, and I could not attend as many sessions as I wanted. This paper is therefore not based on a representative, but rather on a highly selective sample of keynotes, symposiums, oral papers and posters presented at the conference [1]. Nevertheless, even this narrow selection of presentations that I was able to attend, indicates that our annual meeting covered what ISBNPA is all about: *International *focus, a *Society of researchers*, with a focus on *Behavioral research*, related to *Nutrition and Physical Activity*.

## Discussion

### International

The sixth ISBNPA annual meeting reflected the growing internationality of the society. Scientists from more than 30 countries presented their research. However, the vast majority of presentations were from a more selective number of countries and regions, North America, Australia and North-Western Europe in particular. Many countries outside these regions are confronted with similar important behavioral nutrition and physical activity issues and it is a challenge for ISBNPA to create even better opportunities to share our research with colleagues from other countries and to learn from their studies. The fact that some colleagues from Latin America were present at the conference is an important step towards a broader international focus. In other parts of the world issues such as the fast changing nutrition and physical activity patterns, introduction of processed foods, and remaining high prevalence of under-nutrition, combined with a fast growing obesity problem require high quality behavioral research and researchers to monitor and explain behavioral patterns, and inform intervention and policy development. In this respect, it was promising to meet researchers from Africa at the conference, facilitated by a special grant program offered by the Norwegian Directorate for Health and Social Affairs. Countries not represented at the conference, such as many Eastern Asian countries as well as India, have shown very impressive economic growth in recent decades or years combined with similar growth rates in obesity and diabetes prevalence, induced by higher intakes of calories and saturated fat and lower physical activity levels. The situation in China may be illustrative. Although the prevalence of obesity in China is relatively low compared with many Western countries, it is the rapid increase of overweight, especially among children, that is of great concern. Data from national surveys among school children show that the prevalence of overweight and obesity in children and adolescents aged 7–18 years increased 28 times between 1985 and 2000 [2]. The East-Asia regions also offer interesting intervention examples. In Singapore, for example, a school-based obesity prevention program in which health education efforts were combined with strict regulations about food availability at schools and special compulsory exercise programs for overweight and obese students, showed promising effects [3]. It is important to have a broader international exchange within ISBNPA, and we should encourage and facilitate participation of researchers from regions that are now underrepresented.

The international focus of ISBNPA should also be reflected in true international research. The ISBNPA 2007 program included presentations on international comparisons of behavioral nutrition and physical activity issues, such as a symposium on the international renowned Health Behavior in School Children study, a round table session on pan-European European Commission-funded projects and a keynote address by Neville Rigby of the International Obesity task Force on obesity prevention across Europe. Presentations on international research projects, such as those funded by the European Commission's research programs, should be strongly encouraged.

### Society

ISBNPA is not only dedicated to provide a platform to formally present behavioral nutrition and physical activity research, but also to encourage networking and informal contact to encourage and promote exchange of ideas and new research collaborations. The ISBNPA annual meeting in Oslo provided a good platform for networking and discussion. Activities like a keynote debate on the role of governmental regulations to promote healthy nutrition and physical activity, an early investigators meeting mentored by senior scientists of international reputation, the organization of thematic symposiums that allocated time for general discussion and debate, and well-attended receptions for informal exchange of ideas, strengthened the societal character of ISBNPA.

### Behavioral research

ISBNPA's focus is on behavioral research. Nutrition and physical activity are defined by complex combinations of specific behaviors, and these behaviors are influenced by many potential determinants [4]. ISBNPA 2007 confirmed that such behavioral research is a multidisciplinary effort, involving researchers with expertise in nutrition, physical activity, epidemiology, psychology, psychiatry, medicine, sociology, economy, marketing and so on.

Most behavioral nutrition and physical activity research presented at ISBNPA 2007 fits well in the framework of planned promotion of population health [4], covering the steps from the epidemiological analysis to identify which behaviors are associated with health; the analysis of behavioral correlates, predictors or determinants; intervention studies to evaluate efficacy and (cost) effectiveness; and implementation research.

Only few epidemiological studies to identify important specific nutrition and physical activity risk behaviors or preventive behaviors were presented. ISBNPA's research focus is more on describing who engages in such behaviors, explaining why people engage in such behaviors, and using this information to design, test and implement health promotion interventions.

In recent years, behavioral nutrition and physical activity researchers have put much emphasis on the identification of environmental determinants of nutrition, physical activity and sedentary behaviors [5]. This focus on environmental factors in predicting health behavior was certainly present in the ISBNPA 2007 program, with a specific focus on the physical environment and the social environment. These were addressed in different keynotes, symposiums, and other presentations.

Healthful nutrition and physical activity behaviors are unevenly distributed within societies. People with lower socioeconomic positions are more likely to be inactive and eat a less healthy diet [6,7], and it has been suggested that differences in environmental opportunities for health behavior may explain these differences. Professor Sally Mcintyre, from Glasgow University, UK, gave a thought provoking keynote providing evidence that physical environmental opportunities for health behaviors may not differ that much between more advanced and more deprived neighborhoods [8]. She also chaired a symposium on the same topic. The discussant at that symposium, Dr. Frank van Lenthe of Erasmus University Medical Centre, the Netherlands, argued that such differences may vary strongly between countries and specific health behaviors. Other symposiums and presentations also focused on the presumed importance of the physical environment, and it was good to notice that studies now go further than just establishing associations between environmental characteristics and behaviors. A symposium chaired by Professor Ilse de Bourdeaudhuij of Ghent University, Belgium, for example, was dedicated to identify mediators and moderators of relationships between environments and physical activity.

This year's ISBNPA meeting put much emphasis on the potential importance of social environmental influences on nutrition and physical activity behaviors. Professor Leann Birch of Penn State University, USA, gave a comprehensive review of the evidence for family influences on children's eating and physical activity behaviors as well as a research agenda to build a stronger evidence-base. Her review very clearly indicated that present-day insights in such family influences are mostly based on weakly designed studies and that longitudinal and experimental studies are much needed to better guide family-oriented health promotion interventions.

This year's conference did not include many presentations primarily focused on further building or testing health behavioral theories applicable in behavioral nutrition and physical activity research. A notable exception was Professor Richard Ryan's (University of Rochester, USA) keynote on self-regulation theory. Since interventions that build on behavioral theory are more likely to lead to success, further strengthening of theory-driven research should remain one of ISBNPA's goals.

Intervention studies are the real 'proof of the pudding' for promotion of healthful nutrition and physical activity behaviors. While observational studies still constitute the majority of studies presented at ISBNPA, some important intervention studies were included in the program. The ISBNPA program clearly indicated that using information and communication technology in behavioral interventions is very promising and deserves further attention. In symposiums chaired by Professors Tom Baranowski of Baylor College, Austin, USA, Marci Campbell, University of North Carolina at Chapel Hill, USA, and Kim Gans, Brown University, USA, research was presented on such innovative intervention strategies as using computer games, text messaging, podcasting and computer-tailoring to promote healthful nutrition and physical activity.

ISBNPA 2007 also included research on policy interventions. In order to seriously promote more healthful dietary and physical activity habits, isolated, small-scale intervention projects will not be sufficient. Promotion of such behaviors should rather be part of general regional, national and international policies [9]. More restrictive regulations for food advertising to children, especially on TV, is proposed as one policy measure to create a less obesogenic environment. In his keynote lecture on this issue, Dr. Heath McDonald of Deakin University, Australia made very clear that marketing foods to children goes far beyond TV food advertising, that adverting may be the least effective marketing tool, and that restricting TV ads will therefore probably not make much of a difference. The food industry puts much more effort into what is called Integrated Marketing Communications involving such activities as personal selling, competitions, direct marketing and web communications that have a much stronger impact on children's food choice.

The final keynote of ISBNPA 2007 was the keynote debate between Drs. Nick Cavill of the University of Oxford, UK, and Sheila Weiss of the National Restaurant Association, USA. This debate, entitled "Maintaining energy balance: Nanny knows best?", also addressed policy issues: should we strive for a 'Nanny state' where personal choice in behavioral nutrition and physical activity is further restricted by government regulations in order to curb the obesity epidemic? Cavill argued that a certain level of regulation is and has always been an integral part of public health policy, and that the obesity epidemic indeed warrants stronger government-enforced regulations to promote and facilitate healthier choices. Weiss argued that the restaurant industry is already active in providing their customers with healthy choice options and government regulations should not restrict their customers' freedom of choice.

The 2007 ISBNPA conference indicated that behavioral nutrition and physical activity intervention research now goes beyond merely testing if an intervention is effective. First, a symposium chaired by Professor Bess Marcus from Brown Medical School, USA, was dedicated to identify predictors of intervention success, to shed light on why interventions are successful. Such research is needed to identify more general intervention characteristics that promote effectiveness, i.e. to build intervention theory. Second, different presentations addressed implementation of effective interventions. Since true effectiveness of interventions is dependent on efficacy and on reach, it is of great importance to gain more insight in how successful intervention strategies can be disseminated to reach a wider public.

### Nutrition and physical activity research

One of the original purposes of the foundation of ISBNPA was to bring nutrition research and physical activity research together, because both disciplines struggle with the same issues, both behaviors are associated with the same public health problems, and researchers from both fields should and can learn from each other and should join forces to address the important public health challenges [10]. ISBNPA 2007 indicates that we are on the right track to accomplish this integration of research fields. Several symposiums and oral paper sessions integrated nutrition and physical activity issues.

Given the obesity epidemic and its public health consequences, it is not surprising that many presentations at ISBNPA 2007 included research on obesogenic or 'obesopreventive' behaviors. It is good that ISBNPA is concerned with this main determinant of population health in many countries. ISBNPA is, however, not a society for obesity research only. There are many other public health and quality of life issues that involve behavioral nutrition and physical activity issues, such as eating disorders, osteoporosis and other issues related to our aging populations, food safety, behavioral nutrition and physical activity in disease management, or in contributing to cleaner and sustainable environments, to name just a few.

## Conclusion

The 2007 ISBNPA annual meeting in Oslo showed that ISBNPA provides a great platform to present and discuss state-of-the-art behavioral nutrition and physical activity research. ISBNPA lives up to its expectations of being and *International Society *for *Behavioral Nutrition *and *Physical Activity *research. Priorities for future conferences should be to encourage scientists from other regions than North America, Western Europe and Australia to present their work, to encourage longitudinal research and well-designed intervention studies, to maintain our focus on development and testing of behavioral theory, in order to improve our understanding of nutrition, physical activity and health promotion.

## Competing interests

The author declare that they have no competing interests.

## Authors' contributions

JB wrote this paper.
